# Novel nomogram for predicting paradoxical chest wall movement in patients with flail segment of traumatic rib fracture: a retrospective cohort study

**DOI:** 10.1038/s41598-023-47700-w

**Published:** 2023-11-20

**Authors:** Junepill Seok, Soon Tak Jeong, Su Young Yoon, Jin Young Lee, Seheon Kim, Hyunmin Cho, Wu Seong Kang

**Affiliations:** 1 Department of Thoracic and Cardiovascular Surgery, Chungbuk National University Hospital, Cheongju, 28644 South Korea; 2 Department of Physical Medicine and Rehabilitation, Ansanhyo Hospital, Ansan City, Republic of Korea; 3 Department of Trauma Surgery, Chungbuk National University Hospital, Cheongju, 28644 South Korea; 4 Department of Trauma Surgery, Jeju Regional Trauma Center, Cheju Halla General Hospital, 65, Doryeong-ro, Jeju-si, Jeju-do Republic of Korea

**Keywords:** Medical research, Risk factors, Trauma

## Abstract

Flail chest is a severe injury to the chest wall and is related to adverse outcomes. A flail chest is classified as the physiologic, paradoxical motion of a chest wall or flail segment of rib fracture (RFX). We hypothesized that patients with paradoxical chest wall movement would present different clinical features from patients with a flail segment. This retrospective observational study included patients with blunt chest trauma who visited our level 1 trauma center between January 2019 and October 2022 and were diagnosed with one or more flail segments by computed tomography. The primary outcome of our study was a clinically diagnosed visible, paradoxical chest wall motion. We used the least absolute shrinkage and selection operator (LASSO) logistic regression model to minimize overfitting. After a feature selection using the LASSO regression model, we constructed a multivariable logistic regression (MLR) model and nomogram. A total of five risk factors were selected in the LASSO model and applied to the multivariable logistic regression model. Of these, four risk factors were statistically significant: the total number of RFX (adjusted OR [aOR], 1.28; 95% confidence interval [CI], 1.09–1.49; *p* = 0.002), number of segmental RFX including Grade III fractures (aOR, 1.78; 95% CI, 1.14–2.79; *p* = 0.012), laterally located primary fracture lines (aOR, 4.00; 95% CI, 1.69–9.43; *p* = 0.002), and anterior–lateral flail segments (aOR, 4.20; 95% CI, 1.60–10.99; *p* = 0.004). We constructed a nomogram to predict the personalized probability of the flail motion. A novel nomogram was developed in patients with flail segments of traumatic RFX to predict paradoxical chest wall motion. The number of RFX, Grade III segmental RFX, and the location of the RFX were significant risk factors.

## Introduction

Historically, flail chest is a severe injury to the chest wall and is related to adverse outcomes, such as pneumonia, atelectasis, chronic pain, and mortality^1,2^. Flail chest is classified as “physiologic”, based on clinical findings in patients with the paradoxical motion of a chest wall segment, or “anatomical (or flail segment)”, diagnosed by identifying three or more consecutive segmental rib fractures via radiographic examinations such as by computed tomography (CT)^3^. The evolution of CT has especially enabled the accurate diagnosis of flail segments.

The term *flail chest* was initially used to describe the paradoxical motion of a chest wall with difficulty breathing owing to multiple consecutive rib fractures^4^. As the flail segment (an anatomical finding) is a *sine qua non* of the visible flail motion of the chest wall^5^, the term *flail chest* should be clearly distinguished from flail segment^5,6^. Thus, Edward et al. suggested using the term *flail segment* to describe the radiologic appearance and the term *flail chest* to describe the clinical findings^7^. However, only a few studies have followed this classification owing to the complexity associated with the chest wall anatomy, while many others have defined flail chest based solely on the anatomical findings, overlooking the visible flail motion and lacking differentiation between the two^5,6^. Furthermore, while long bone fractures can be immobilized using splints, rib fractures cannot be immobilized unless surgical fixation is performed. Therefore, throughout the healing process, this can lead to an increased degree of displacement, which can potentially result in delayed flail motion^8–11^. Indeed, flail motion has only recently been demonstrated as a significant risk factor for adverse pulmonary outcomes^12^.

We conducted a retrospective study with prospectively recorded data to investigate the risk factors associated with flail chest. We hypothesized that patients with flail motion would present different clinical features from patients with only flail segment and without flail motion We also established a prediction model using a novel nomogram that accounts for risk factors that cause visible flail motions in patients with one or more flail segments.

## Material and methods

### Study design and data source

A retrospective observational single-center study was conducted, with all methods performed in accordance with the relevant guidelines and regulations^13^. This study aimed to establish a prediction model for paradoxical chest wall movements in patients with multiple rib fractures and analyze the risk factors associated with paradoxical chest wall movement. The primary outcome of our study was a clinically diagnosed visible, paradoxical chest wall motion.

The study was conducted at a level 1 trauma center in Chungbuk National University Hospital, Cheongju, Korea. Our institution is a tertiary care university-affiliated hospital with 800 beds, making it one of the biggest trauma centers in South Korea. The hospital is responsible for 2.5 million people, with almost 450 patients presenting with an Injury Severity Score (ISS)^14^  > 15 annually. Informed consent was waived by the IRB at Chungbuk National University Hospital due to the observational nature of the study. We prospectively recorded the data for all patients presenting with blunt chest trauma from the time of admission, including the ISS and Abbreviated Injury Scale (AIS)^15^. Patient progressions were also prospectively recorded, such as the presence of paradoxical chest wall motion or pneumonia during the index hospitalization. The patterns of the rib fractures (RFX) and degree of pulmonary contusions (PC) were recorded once based on the initial chest CT, which was performed by a thoracic surgeon with more than 10 years of experience, and who was affiliated with the trauma center. In our trauma center, CT scans were performed on all patients from the head to the pelvis. CT scans were also performed on the extremities if required. All patient data were encoded to ensure the privacy of the subjects and data confidentiality.

To ensure the accuracy and reliability of the findings, all assessments were subsequently validated during daily multidisciplinary meetings by a radiologist and other trauma surgeons. These meetings served as a comprehensive platform to review and discuss each hospitalized patient, including those newly admitted on the previous day. The evaluation of flail motion, a crucial aspect of the study, was also carried out by the experienced thoracic surgeon. In instances where the thoracic surgeon was unavailable to perform the assessment, recorded video footage was used to analyze and determine the presence of the flail motion. However, in cases where consent for the video recording was not obtained or when a video recording was not feasible, the thoracic surgeon closely observed and assessed the movement of the chest wall over a span of two hospital days.

### Study population, definitions, and inclusion and exclusion criteria

**Figure 1:**
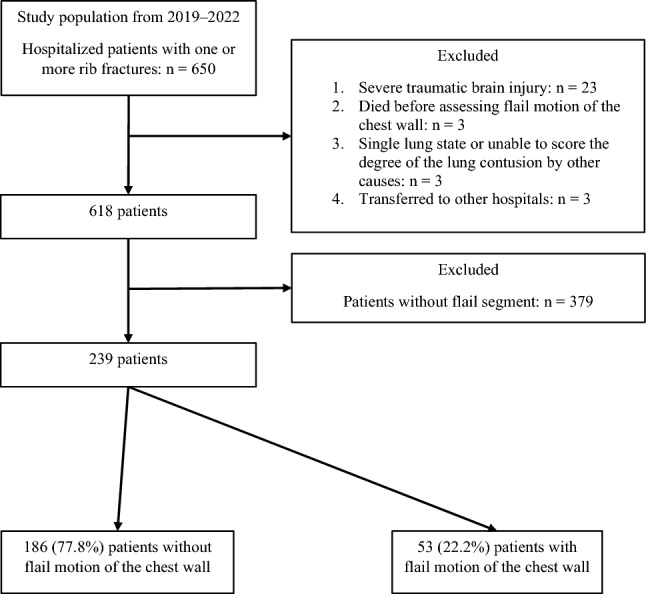
Flow chart for patient selection.

This study enrolled consecutive patients with blunt chest trauma who presented to our trauma center's emergency department (ED) between January 2019 and October 2022. The cohort in this study excluded patients who did not survive at the ED. Additional exclusion criteria included: (a) severe traumatic brain injury of AIS head > 3 since it may affect respiratory function; (b) death while on a mechanical ventilator before an assessment of the self-movement of the chest wall; (c) conditions in which the degree of PC could not be assessed, such as a collapsed lung due to tension pneumothorax or one lung state due to a previous history of pneumonectomy; (d) transfer to other hospitals within 24 h of presentation; (e) patients without a flail segment (Fig. 1).

We classified the RFX patterns into three broad categories: the number of RFX, the RFX locations, and the type of flail segment.

#### Number of RFX with the degree of displacement

We calculated the number of ribs using the degree of fracture displacement. Currently, there are two major discussions or bases regarding the classification of rib fractures. Previously, Chien et al.^16^ and various other studies have suggested a classification whereby fractures are divided into Grade I and Grade II based on a 50% displacement threshold (Grade 0: no rib fractures; Grade I: rib fractures with a displacement of < 50% of rib the width on axial CT; Grade II: between > 50% and < 100%), while fractures that are completely dislocated are classified as Grade III. Recently, Edwards et al.^7^ have suggested another classification whereby fractures are divided into “Undisplaced”, “Offset” based on a 10% displacement threshold (Undisplaced: rib fractures with a displacement of < 10% of the rib width on axial CT; Offset: between > 10% and < 100%), while fractures that are completely dislocated are classified as “Displaced”. In our study, we applied both of these criteria and derived the results accordingly. [ref]^7,17,18^ Additionally, even if a single rib was fractured into two or more pieces, only the fractures at the two most severely broken locations were recorded and evaluated. Thereafter, based on these scores, we calculated the variables described below.

#### RFX Locations

The rib fracture location was divided into three parts using the anterior and posterior axillary lines (Supplementary Fig. 1)^7^. However, the upper 1st–2nd ribs and lower 11th–12th ribs did not follow the anatomic landmarks. Therefore, in our raw database, these cases were recorded by drawing imaginary lines that coincided with the landmarks of the 3rd–10th ribs.

We introduced a new concept of a “primary fracture line” to represent the RFX patterns in more detail (Supplementary Fig. 2). Rib fractures, including flail segments encompassing the posterior part, tend to be better tolerated than anterior or lateral injuries due to the surrounding structures, such as the scapula, providing stability^19,20^. We hypothesized that even patients with the same type of flail segment would show different clinical features depending on whether the lateral or posterior fractures were more severe and that the RFX locations would prove meaningful when grouped. Clinically, multiple rib fractures tend to occur perpendicular to the ribs and form a relatively straight line. A flail segment may have at least two straight fracture lines. If two fracture lines were identified in a patient with an ipsilateral flail segment of the chest wall, a fracture line consisting of more severely dislocated ribs was defined as the primary fracture line, and its anatomical location was recorded, i.e., the anterior line, lateral line, and posterior line. Thus, most patients with ipsilateral multiple rib fractures could have one primary fracture line, and patients with bilateral multiple rib fractures could have up to two primary fracture lines (each per hemithorax).

#### Types of the flail segment

A segmental rib fracture was diagnosed when a single rib had ≥ 2 fractures at different locations. As the lateral part is the most extended among the anterior, lateral, and posterior anatomical parts, lateral–lateral types in the segmental fractures were present in some patients. Moreover, three patients had flail segments each consisting of ipsilateral multiple segmental rib fractures on the costal cartilages and costochondral junctions. These cases were defined as ipsilateral anterior–anterior flail segments.

Flail chest was subclassified and defined as follows: (a) anatomical flail segment: radiologically confirmed two or more consecutive segmental rib fractures^21^; (b) flail motion: clinically confirmed paradoxical movement of the chest wall during the index hospitalization. Additionally, a flail segment consisting of bilateral multiple rib fractures on the anterior portions of each hemithorax with or without sternal fracture was defined as a bilateral anterior–anterior flail segment. If a flail segment consisted of two or more consecutive segmental fractures that were located in totally different portions, it was defined as indistinguishable. Thus, this study described seven types of flail segments: type 1: bilateral anterior–anterior; type 2: ipsilateral anterior–anterior; type 3: ipsilateral anterior–lateral; type 4: ipsilateral anterior–posterior; type 5: ipsilateral lateral–lateral; type 6: ipsilateral lateral–posterior; type 7: indistinguishable. Similar to the primary fracture line, patients with bilateral RFX could have multiple flail segment types.

Among 12 pairs of ribs, the upper two ribs (1st–2nd) and lower two ribs (11–12th) are relatively less important in the respiratory function^20,22,23^. Therefore, we excluded those four ribs when defining the flail segment and established equations using other ribs (3rd-10th) to calculate the flail segment and primary fracture line locations.

Moreover, the degree of PC was scored using the blunt pulmonary contusion score (BPC18)^24,25^, which divides each lung field into the upper, middle, and lower third. Each third received a score of 0 to 3 based on the density of the affected lung. Further, we calculated the degree of chest trauma using various scoring systems, such as Thorax Trauma Severity Score (TTSS)^26^, Rib Fracture Score (RFS)^27^, Chest Trauma Score (CTS)^28^, and RibScore^29^ (Supplementary Table 1).

### Statistical analysis

The median and interquartile range (IQR) are used to represent the continuous data, while proportions are used to represent the categorical data. Continuous data were compared using Student’s t-test or Mann–Whitney U test. Proportions were compared using the Chi-square or Fisher’s exact tests, as appropriate. Significance was set at *p* < 0.05. All statistical analyses were conducted using the R language, version 4.1.2 (R foundation, Vienna, Austria)^30^. We used the “autoReg^31^,” “multipleROC^32^,” “glmnet^33^,” “tidyverse^34^,” “rms^35^,” and “patchwork^36^” packages for data analysis and visualization.

To minimize the prediction model's overfitting and enhance the accuracy of the new dataset, we used the least absolute shrinkage and selection operator (LASSO) to reduce the regression coefficients to zero^37,38^. We performed a tenfold cross-validation to select the optimal hyperparameter (λ). In the cross-validation, optimal λ was chosen as the most regularized model so that the error was within one standard error of the minimum^37^. We input risk factors into the LASSO regression model for the flail motion, including age, sex, body mass index, bilaterality of rib fracture, number of rib fractures (simple + segmental or segmental), primary fracture line location, flail segment type, and sternal fracture.

After conducting feature selection using the LASSO regression model, we constructed a multivariable logistic regression (MLR) model. Based on the logistic regression model, we delineated a nomogram, which is a graphical calculating device that allows approximate probability computation^39^. We used a receiver operator characteristic (ROC) curve to evaluate the performance of the prediction model and calculate the area under the ROC curve (AUROC)^28^. Finally, we used Youden’s index to calculate the optimal cut-off value^40^.

## Results

### Patient characteristics

The study population, including the inclusion and exclusion criteria, is delineated in Fig. 1. Table 1 presents the baseline characteristics and outcomes of the study population. During the study period, 239 patients were included who possessed one or more flail segments. We divided the patients into two groups: those with or without flail motion in the chest wall. As a result, 186 (77.8%) patients only possessed an anatomical flail segment, while 53 (22.2%) had both the flail segment and flail motion.

**Table 1:** Comparison of clinical characteristics and outcomes of patients with one or more flail segments.

	Total	Without flail motion	With flail motion	*p*
	(N = 239)	(N = 186, 77.8%)	(N = 53, 22.2%)	
Sex, n (%)				1.000
Female	67 (28.0%)	52 (28.0%)	15 (28.3%)	
Male	172 (72.0%)	134 (72.0%)	38 (71.7%)	
Age, median [IQR]	61.0 [53.0–71.0]	60.0 [51.0–70.0]	67.0 [58.0–73.0]	0.022*
BMI, median [IQR]	24.0 [22.0–26.6]	24.0 [22.1–26.7]	23.9 [21.9–26.0]	0.417
BPC18, median [IQR]	2.0 [1.0–5.0]	2.0 [1.0–4.0]	4.0 [1.0–8.0]	0.001*
Initial PFR, median [IQR]	282.5 [188.8–347.8]	301.1 [213.0–354.8]	216.2 [94.7–317.4]	< 0.001*
Length of stay, median [IQR]				
Hospital LOS, day	19.0 [11.0–34.0]	16.0 [9.0–30.0]	31.0 [20.0–48.0]	< 0.001*
ICU LOS, min	2910.0 [0.0–8177.5]	1802.5 [0.0–5310.0]	9170.0 [3825.0–23,760.0]	< 0.001*
MV LOS, min	0.0 [0.0–1002.5]	0.0 [0.0–0.0]	1960.0 [0.0–12,500.0]	< 0.001*
Pneumothorax, n (%)	177 (74.1%)	137 (73.7%)	40 (75.5%)	0.930
Hemothorax, n (%)	188 (78.7%)	143 (76.9%)	45 (84.9%)	0.286
Pulmonary complications				
Pneumonia	54 (22.6%)	22 (11.8%)	32 (60.4%)	< 0.001*
MV > 48 h	40 (16.7%)	16 (8.6%)	24 (45.3%)	< 0.001*
Tracheostomy	13 (5.4%)	4 (2.2%)	9 (17.0%)	< 0.001*
Other surgical complications	10 (4.2%)	5 (2.7%)	5 (9.4%)	0.076
RFX patterns: no. of RFX, median [IQR]				
Total (simple + segmental)	7.0 [6.0–10.0]	7.0 [6.0–9.0]	11.0 [7.0–13.0]	< 0.001*
Grade I	4.0 [2.0–6.0]	4.0 [2.0–6.0]	3.0 [2.0–6.0]	0.945
Grade II	1.0 [0.0–2.0]	1.0 [0.0–2.0]	1.0 [0.0–3.0]	0.182
Grade III	2.0 [1.0–4.0]	2.0 [1.0–3.0]	5.0 [3.0–7.0]	< 0.001*
Segmental	4.0 [3.0–5.0]	3.0 [2.0–4.0]	5.0 [3.0–7.0]	< 0.001*
Grade I	1.0 [0.0–2.0]	1.0 [0.0–2.0]	1.0 [0.0–2.0]	0.272
Grade II	0.0 [0.0–1.0]	0.0 [0.0–1.0]	0.0 [0.0–1.0]	0.507
Grade III	2.0 [0.0–3.0]	1.0 [0.0–2.0]	3.0 [2.0–5.0]	< 0.001*
RFX patterns: Primary fracture line locations, n (%)				
Anterior line	53 (22.2%)	41 (22.0%)	12 (22.6%)	1.000
Lateral line	115 (48.1%)	75 (40.3%)	40 (75.5%)	< 0.001*
Posterior line	94 (39.3%)	82 (44.1%)	12 (22.6%)	0.008*
RFX patterns: Flail segment types, n (%)				
Bilateral ant.––ant	50 (20.9%)	30 (16.1%)	20 (37.7%)	0.001*
Ipsilateral ant.––ant	3 (1.3%)	2 (1.1%)	1 (1.9%)	1.000
Ant.––Lat	36 (15.1%)	20 (10.8%)	16 (30.2%)	0.001*
Ant.––Post	17 (7.1%)	15 (8.1%)	2 (3.8%)	0.442
Lat.––Lat	7 (2.9%)	5 (2.7%)	2 (3.8%)	1.000
Lat.––Post	150 (62.8%)	120 (64.5%)	30 (56.6%)	0.373
Indistinguishable	7 (2.9%)	6 (3.2%)	1 (1.9%)	0.961
Bilateral RFX	67 (28.0%)	40 (21.5%)	27 (50.9%)	< 0.001*
Sternal fracture	4 (1.7%)	4 (2.2%)	0 (0.0%)	0.639
Scoring systems, median [IQR]				
ISS	17.0 [13.0–24.0]	17.0 [13.0–22.0]	20.0 [17.0–30.0]	< 0.001*
AIS head	0.0 [0.0–2.0]	0.0 [0.0–0.0]	0.0 [0.0–2.0]	0.041*
AIS face	0.0 [0.0–0.0]	0.0 [0.0–0.0]	0.0 [0.0–0.0]	0.089
AIS chest	3.0 [3.0–3.0]	3.0 [3.0–3.0]	3.0 [3.0–4.0]	< 0.001*
AIS abdomen	0.0 [0.0–2.0]	0.0 [0.0–2.0]	2.0 [0.0–3.0]	0.005*
AIS extremities	2.0 [0.0–2.0]	2.0 [0.0–2.0]	2.0 [0.0–3.0]	0.015*
AIS external	1.0 [0.0–1.0]	1.0 [0.0–1.0]	1.0 [0.0–1.0]	0.349
TTSS	13.0 [11.0–15.0]	12.0 [10.0–14.0]	16.0 [13.0–18.0]	< 0.001*
RFS	10.0 [7.0–16.5]	9.0 [7.0–12.0]	18.0 [9.0–27.0]	< 0.001*
CTS	7.0 [6.0–8.0]	7.0 [6.0–8.0]	8.0 [7.0–9.0]	< 0.001*
RibScore	3.0 [2.0–4.0]	3.0 [2.0–4.0]	4.0 [3.0–5.0]	< 0.001*
SSRF	35 (14.6%)	5 (2.7%)	30 (56.6%)	< 0.001*

n and no.: number; min: minutes; BMI: body mass index; IQR: interquartile ranges; BPC18: Blunt Pulmonary Contusion score; PFR: PaO2/FiO2 ratio; ICU: intensive care unit; MV: mechanical ventilator; LOS: length of stay; RFX: rib fractures; Ant.: anterior; Lat.: lateral; Post.: posterior; ISS: Injury Severity Score; AIS: Abbreviated Injury Scale; TTSS: thoracic trauma severity score; RFS: rib fracture score; CTS: chest trauma score; SSRF: surgical stabilization of rib fractures.

*: statistical significance when *p* < 0.05,

Compared to the non-flail motion group, the flail motion group showed a longer length of stay (LOS) in hospital (hospital LOS, median, [IQR]: 16.0, [9.0–30.0] vs. 31.0, [20.0–48.0]; intensive care unit LOS, median, [IQR]: 1802.5, [0.0–5310.0] vs. 9170.0, [3825.0–23,760.0]; duration on the mechanical ventilator LOS, median, [IQR]: 0.0, [0.0–0.0] vs. 1960.0, [0.0 -12,500.0], *p* < 0.001, respectively). Among the 186 patients who did not exhibit flail motion, 30 received mechanical ventilator support. However, interquartile range analysis found that both Q1 (lower 25%) and Q3 (upper 25%) within this group had values of 0 for the duration of mechanical ventilator support, meaning all 30 patients who received mechanical ventilator support were considered outliers. Pulmonary complications that were more frequently observed in the flail motion group included pneumonia (11.8 vs. 60.4%, *p* < 0.001), prolonged duration (more than 48 h) on the mechanical ventilator (8.6 vs. 45.3%, *p* < 0.001), tracheostomy (2.2 vs. 17.0%, *p* < 0.001), and other pulmonary complications, such as empyema, which needed surgical management (2.7 vs. 9.4%, *p* < 0.001).

**Figure 2:**
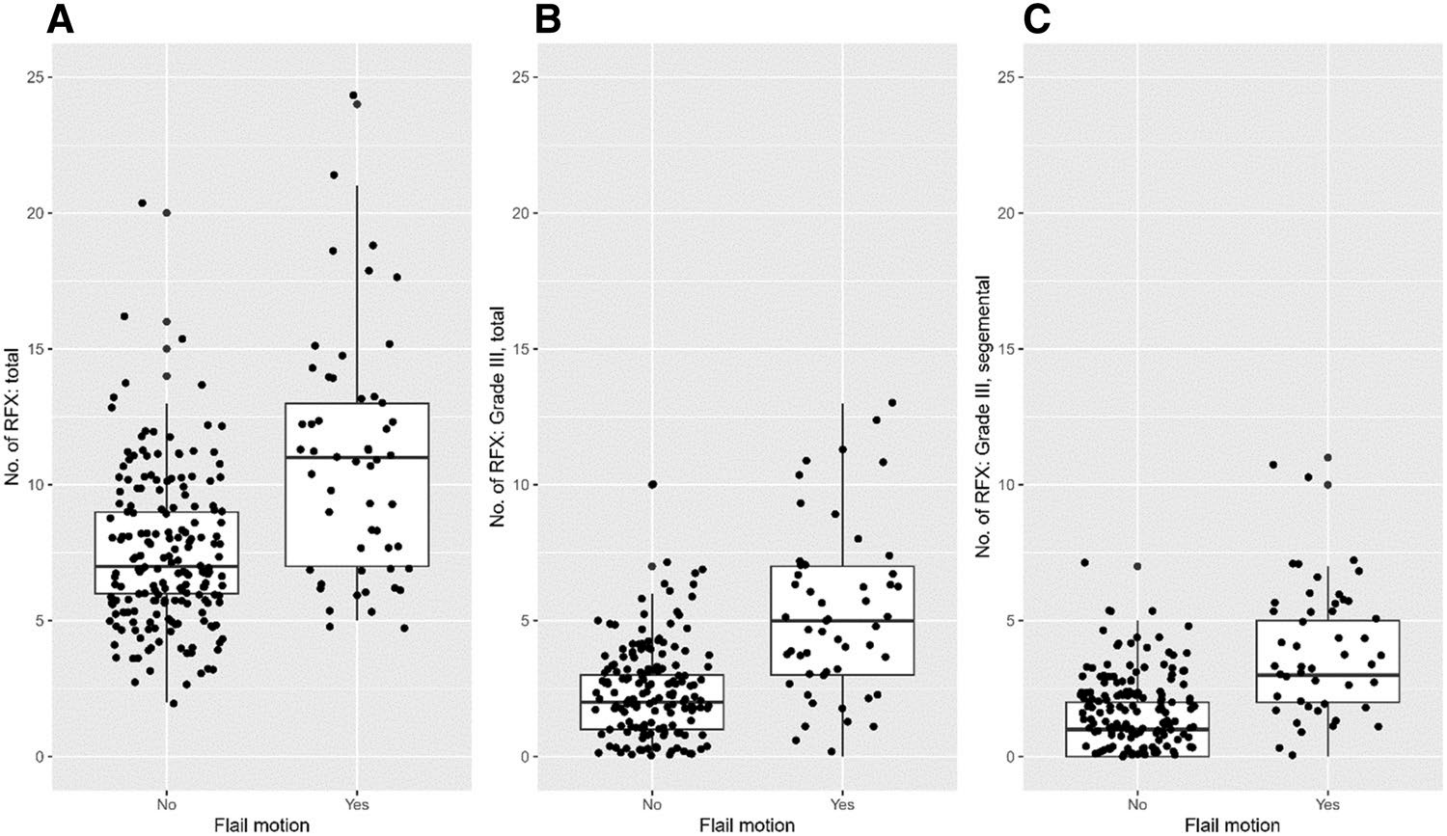
Boxplots of the fracture patterns. (**A**) total number of fractured ribs (simple + segmental); (**B**) total number of fractured ribs with Grade III displacement (simple + segmental); (**C**) number of segmental rib fractures with Grade III displacement.

RFX patterns were significantly different between both groups. The flail motion group had more total rib fractures (7.0, [6.0–9.0] vs. 11.0, [7.0–13.0], *p* < 0.001). Specifically, the total number of Grade III RFX was significantly different (2.0, [1.0–3.0] vs. 5.0, [3.0–7.0], *p* < 0.001). However, there was no significant difference between the number of Grades I and Grade II RFX (4.0, [2.0–6.0] vs. 3.0, [2.0–6.0], *p* = 0.945; 1.0, [0.0–2.0] vs. 1.0, [0.0–3.0], *p* = 0.182, respectively). Even when limited to segmental fractures, the flail motion group showed more severe patterns (total number of segmental RFX: 3.0, [2.0–4.0] vs. 5.0, [3.0–7.0], *p* < 0.001; the number of segmental RFX including Grade III fractures: 1.0, [0.0–2.0] vs. 3.0, [2.0–5.0], *p* < 0.001) (Fig. 2).

The locational variables of RFX patterns were notably different between the two groups. The non-flail motion group tended to have more severe fractures posteriorly (primary fracture line: 44.1 vs. 22.6%, *p* = 0.008), while the flail motion group tended to have more severe fractures in the lateral portion (primary fracture line: 75.5 vs. 40.3%, *p* < 0.001).

Among the seven types of flail segment, the bilateral anterior–anterior and anterior–lateral types were statistically different (16.1 vs. 37.7%; 10.8 vs. 30.2%, *p* = 0.001, respectively). The flail motion group also had more patients with bilateral RFX (21.5 vs. 50.9%, *p* < 0.001).

In addition to RFX patterns, the flail motion group showed the worst scores in most scoring systems, including ISS, TTSS, RFX, CTS, and RibScore (*p* < 0.001).

### Risk factor analysis using the LASSO regression model

**Figure 3:**
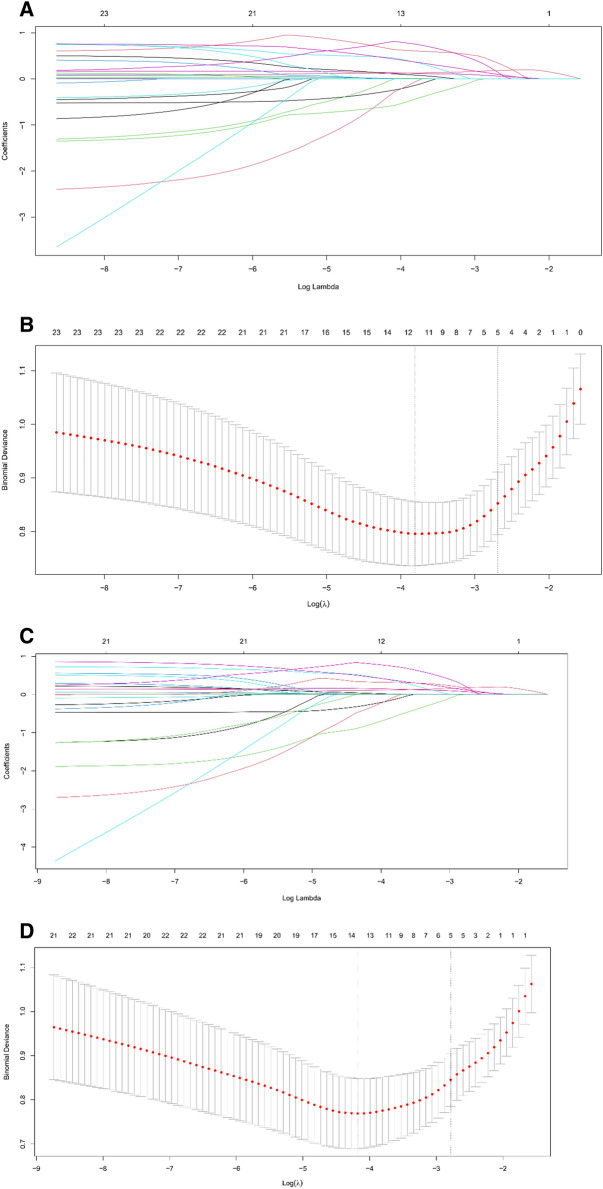
Clinical variables were selected using the LASSO logistic regression model alongside the rib fracture Grade I–III scoring system (**A** and **B**) and “undisplaced”, “offset”, and “displaced” nomenclature (**C** and **D**). (**A**) Shrinkage of coefficients by hyperparameter (λ). (**B**) Hyperparameter selection (λ) using cross-validation. The dotted line indicates the value of the harmonic log (λ) when the model error is minimized. In the LASSO logistic regression model, five variables were selected when log (λ) was -2.6902. (**C**) Shrinkage of coefficients by hyperparameter (λ). (**D**) Hyperparameter selection (λ) using cross-validation. The dotted line indicates the value of the harmonic log (λ) when the model error is minimized. In the LASSO logistic regression model, five variables were selected when log (λ) was -2.7832.

Analysis using the LASSO logistic regression model is summarized in Fig. 3. Figure 3A delineates the shrinkage of coefficients by the hyperparameter (λ), and Fig. 3B delineates the model's accuracy via cross-validation. In the cross-validation, the optimal log (λ) was − 2.6902. At this level, the five selected risk factors were the total number of RFX (simple + segmental), the total number of fractured ribs including Grade III displacement (simple + segmental), the number of segmental RFX including Grade III fractures, the primary fracture line located in the lateral portion, and the anterior–lateral type of the flail segment. The LASSO shrank the coefficient estimates of the other risk factors toward zero. Using “undisplaced, “offset”, and “displaced” in the analysis nomenclature, the five selected risk factors were the total number of RFX (simple + segmental), the total number of fractured ribs including “displaced” for displacement (simple + segmental), the number of segmental RFX including “displaced” fractures, the primary fracture line located in the lateral portion, and the anterior–lateral type of flail segment in LASSO logistic regression model (Fig. 3). Thus, the same MLR model and nomogram were formulated because “displaced” is identical to Grade III displacement.

### Prediction model, nomogram, and model performance

**Figure 4:**
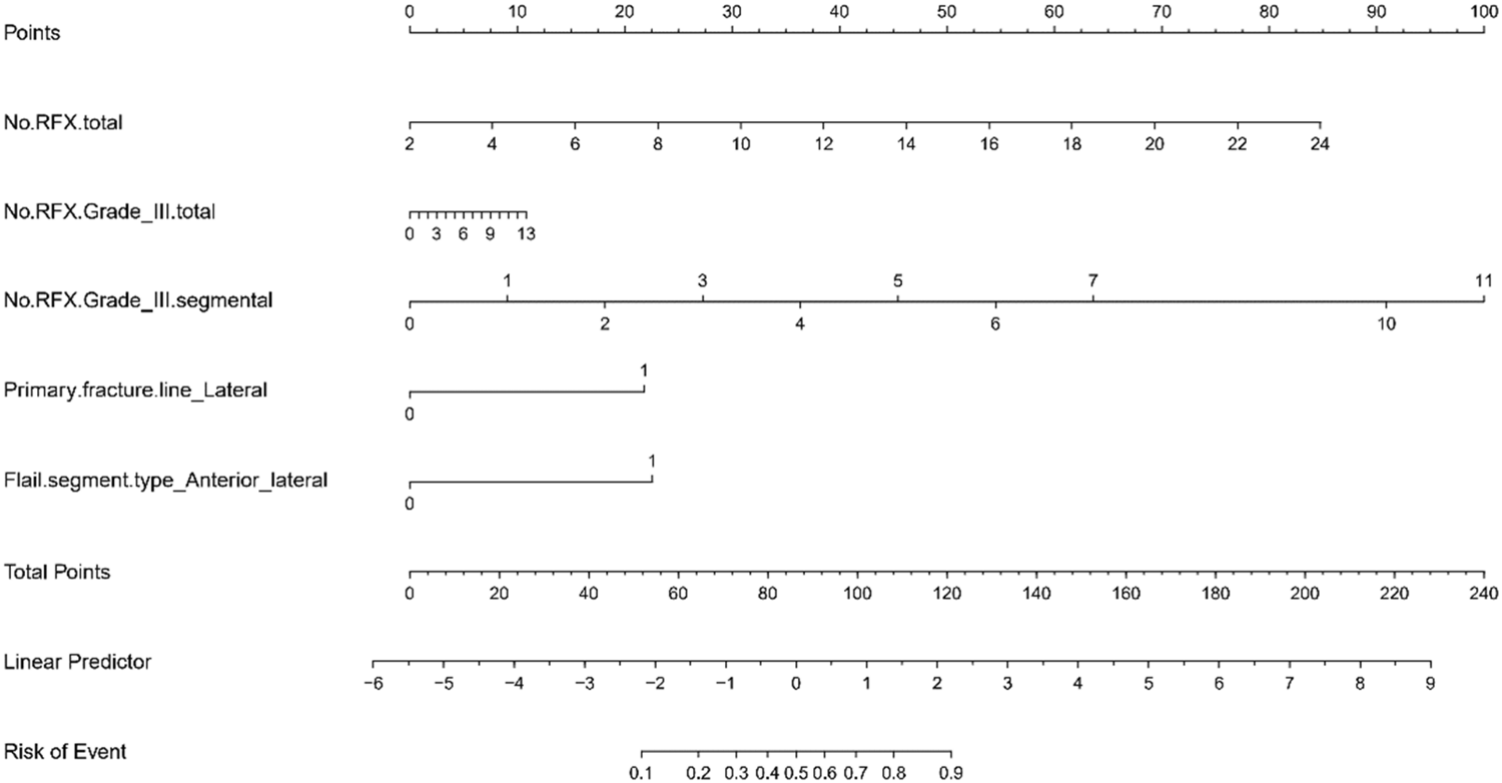
Nomogram predicts the risk associated with flail motion. Each variable is assigned a score on each axis. The sum of all points for all variables is computed and denoted as the total points. The predicted probability can be obtained on the lowest row corresponding to the sum of total points.

**Figure 5:**
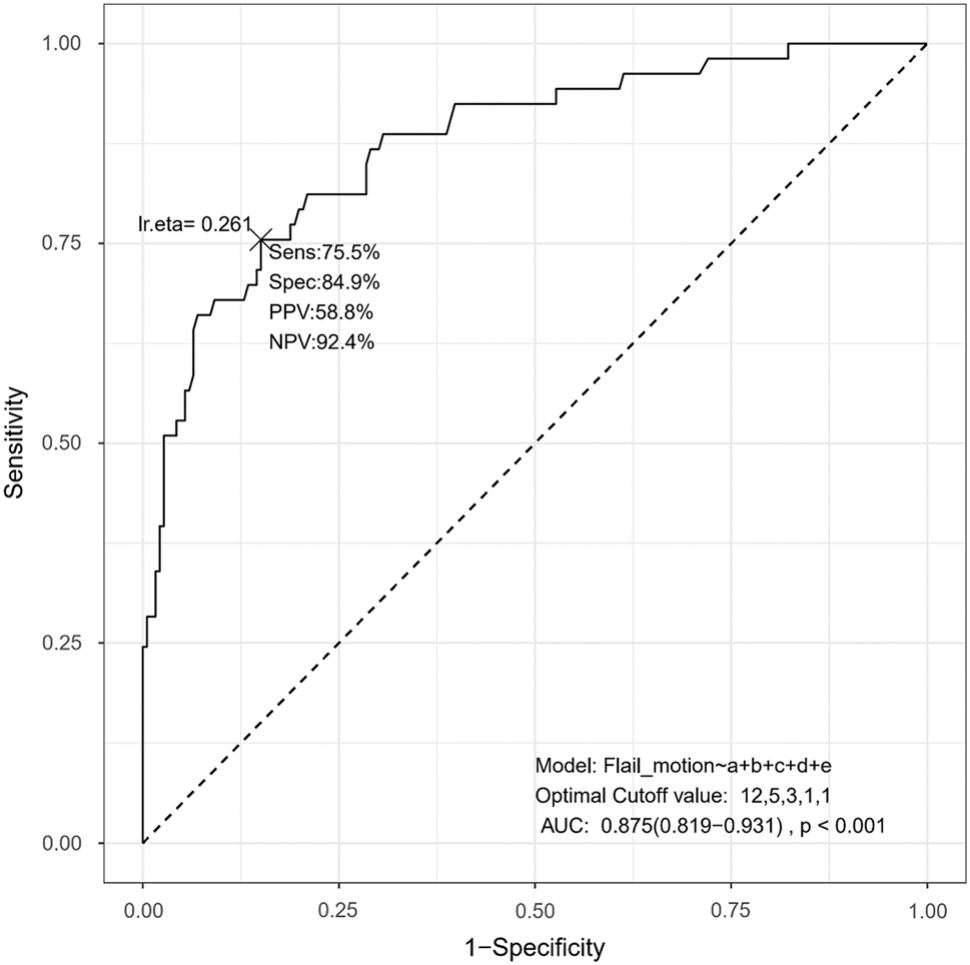
The accuracy of a multivariable logistic regression model for predicting flail motion. (**a**): total number of fractured ribs (simple + segmental); (**b**): total number of fractured ribs with Grade III displacement (simple + segmental); (**c**): number of segmental rib fractures with Grade III displacement; (**d**): laterally located primary fracture line; (**e**): the anterior-lateral type of flail segment.

The MLR model that used the five risk factors selected by the LASSO model is summarized in Table 2. In the MLR analysis, the total number of RFX (adjusted odds ratio [aOR], 1.28; 95% confidence interval [CI], 1.09–1.49; *p* = 0.002), the number of segmental RFX including Grade III fractures (aOR, 1.78; 95% CI, 1.14–2.79; *p* = 0.012), laterally located primary fracture line (aOR, 4.00; 95% CI, 1.69–9.43; *p* = 0.002), and the anterior–lateral type of flail segment (aOR, 4.20; 95% CI, 1.60–10.99; *p* = 0.004) were statistically significant. The total number of fractured ribs, including Grade III displacement (simple + segmental), was selected in the LASSO logistic regression mode, although it did not show statistical significance in the MLR model (*p* = 0.773). We constructed a nomogram to predict the personalized probability of the flail motion in the chest wall (Fig. 4). The AUROC in our proposed model was 0.875 (Fig. 5). The optimal cut-off values for these five variables are as follows: the total number of fractured ribs (simple + segmental): 12; the total number of fractured ribs with Grade III displacement (simple + segmental): 5; the total number of segmental rib fractures with Grade III displacement: 3; the existence of the primary fracture line located in the lateral part; the existence of the anterior–lateral type of flail segment. At the cut-off value, our model showed a sensitivity of 75.6%, a specificity of 84.9%, a positive predictive value of 58.8%, and a negative predictive value of 92.4%.

**Table 2:** Univariable and multivariable analyses of risk factors for flail motion of the chest wall.

	Multivariable			
	aOR	95% CI of aOR		*P*
		Lower	Upper	
No. of RFX: total	1.28	1.09–	1.49	0.002
No. of RFX: Grade III, total	1.05	0.74–	1.51	0.773
No. of RFX: Grade III, segmented	1.78	1.14–	2.79	0.012
Primary fracture line: Lateral	4.00	1.69–	9.43	0.002
Flail segment type: Anterior-lateral	4.20	1.60–	10.99	0.004

aOR: adjusted odd ratio; CI: confidence interval; RFX: rib fracture; No.: number.

### Institutional review board statement

This study was approved by the institutional review board of the Hospital (IRB no. CBNUH 2023–03-024). Informed consent was waived due to the study's observational nature.

## Discussion

Our study was conducted on patients with one or more flail segments, whereby patients with flail motion showed worse clinical features than patients without flail motion in terms of the length of the hospital stay, length of ICU stay, duration of mechanical ventilation, pneumonia, tracheostomy, and other surgical complications. The paradoxical chest movement per se appeared to be the most severe phenotype associated with multiple rib fractures. Our study suggests a prediction model, which can be visualized by a novel nomogram to predict paradoxical chest wall motion in patients with flail segments that have occurred by blunt trauma. To the best of our knowledge, our study is the first to investigate this issue. Our study also showed that the flail segment is a necessary but not a sufficient condition for flail motion in the chest wall and that the flail segment with flail motion is the most severe type of chest injury. Getz et al. suggested distinguishing between flail segment and flail motion^5^. Many studies that did not distinguish between flail segment and flail motion showed uncertain results on the clinical value of flail chest^5,6,41–43^. However, the clinical manifestations between the two groups were significantly different. Our study prospectively recorded all patient data to identify patients with delayed-onset flail chest motion during the index hospitalization and successfully divided patients into two groups.

To identify the significance of the location of rib fractures, only some authors have suggested that RFX on the posterior chest wall might be better tolerated than on the anterior or lateral portions^20,44^. In our study, two risk factors for flail motion support those opinions. Our study introduced a novel concept of “primary fracture line” for the first time and calculated the validity of the concept. Thus, we found that not all flail segments of the same type show the same clinical manifestations. In our study, among patients with the same posterolateral flail segment, patients with more severely broken ribs in the lateral part had a higher probability of visible flail motion than others that were located in the posterior. Furthermore, the anterior–lateral flail segment type showed higher probabilities for flail motion than other types. However, the posterior rib fractures showed a relatively minor influence on flail motion. Most trauma patients tend to stay bedridden and receive treatments at the beginning of hospitalization. For posterior rib fractures, in addition to the support of the surrounding structures, including the scapula mentioned above, the patient’s bed mattress acts as a cushion, thus, securing relatively better stability than in the anterior or lateral fractures. However, the evidence regarding this issue remains limited, although our results appear remarkable.

Our study identified 262 primary fracture lines and 270 flail segments in 239 patients. The 239 patients had a total of 1955 fractured ribs. According to the Grade I, II, and III classification criteria, 978 fractured ribs were categorized as Grade I, 301 were Grade II, and 676 were Grade III, while 618, 661, and 676 were classified as “undisplaced”, “offset”, and “displaced”, respectively. However, in our study, the results indicated that both Grade I and Grade II, as well as the “undisplaced” and “offset” criteria, were not statistically significant. Conversely, statistical significance was observed in the "complete displacement" category for both classification criteria. Therefore, we believe that future studies with larger sample sizes will potentially detect more suitable classification criteria.

In the univariate analysis of our study, several variables, such as the bilateral anterior–anterior flail segment and the total number of fractured ribs with Grade III (simple + segmental), were found to be statistically significant risk factors for flail motion. However, these variables were excluded from the final model following LASSO regression regularization. Future large-scale studies are needed to address this issue.

Our study suggested that the total number of RFX and the number of segmental RFX with Grade III displacement were significant risk factors for flail motion in the chest wall. Several previous studies^26–29^ reported that the number of RFX, with or without displacement, was related to adverse clinical outcomes. Although the primary outcome differed from ours, our results show relevance with previous research, whereby the flail motion group in our study showed more pulmonary complications in the univariate analyses than the non-flail group. Future studies should address in more detail the role of the flail motion in the chest wall for the adverse outcomes.

Recently, the Chest Wall Injury Society conducted a Delphi consensus exercise that addressed multiple rib taxonomic issues, including fracture location, degree of displacement, associated fractures in neighboring ribs, and the definition of a flail chest^7^. In addition to this consensus, we excluded four ribs (1st, 2nd, 11th, and 12th) from the equation while calculating the locational patterns of RFX. Common sense is that the lower two ribs— “floating ribs” —rarely contribute to the respiratory function of the chest wall. Moreover, the 1st rib is related to the spinal cord, carotid artery, or head injuries, and is not a hallmark of chest trauma^45–47^. Thus, our study suggested detailed RFX patterns as risk factors for flail motion, including the number, location, and type of flail segment, all of which have never been reported.

As mentioned, we defined the flail segment as two or more consecutive segmental RFX, confined to the 3rd–10th ribs. By this definition, among the 650 patients primarily enrolled during the study period, 239 patients exhibiting at least one flail segment were finally included. Among the 411 excluded patients without a flail segment, nobody showed flail motion in the chest wall except for one patient. The patient was a victim of an industrial accident and was struck in the anterior chest wall by a large H-beam. The patient presented with paradoxical chest wall motion consisting of multiple rib fractures as follows: the right 1st rib: a segmental fracture on the anterior and the posterior parts, both were Grade III; the right 2nd rib: a simple fracture on the lateral part, Grade II; the left 2nd–6th multiple rib fractures, all were on the costal cartilages, and all were Grade I. The patient also had a sternal body fracture and an oblique shape with total dislocation. However, the patient’s RFX pattern could not satisfy the definition of a flail segment and they were considered in the non-flail segment group. Therefore, further discussion on the role of the 1st and 2nd ribs in blunt chest trauma is necessary.

We believe that by using the model presented in our study, the need for surgical rib fixation can be more quickly determined in patients receiving induced sedation for traumatic brain injury, spinal injury, or abdominal injury. Furthermore, we believe that the proposed model can enhance patient safety by providing indications for early surgical stabilization in cases of multiple rib fractures with the potential for delayed flail motion.

Our study has several limitations. First, the retrospective design may induce selection bias, whereby the excluded patients may have severe rib fractures that are classified as the most severe form. To address this issue, a larger prospective study is warranted. Second, the RFX patterns were recorded once based on the initial chest CT. As the degree of RFX displacement changes over time^8^, a follow-up with a repeat chest CT was necessary; however, we could not perform chest CT scans due to cost and patient safety. Third, the study included a small number of patients who underwent SSRF. Although surgical fixation might affect the patient’s prognosis, our principle of SSRF is very conservative. We rarely perform SSRF with patients who do not show flail motion in the chest wall. Our surgical indication for SSRF is for patients with difficulty breathing with visible flail motion and for the “on the way out” procedure after thoracotomy for another indication. We do not perform SSRF to prevent probable respiratory complications. Hence, the probability that SSRF might lower the incidence of flail motion would be less likely in our study. The authors hope that this study may contribute in some way to establishing indications for surgical stabilization of rib fractures. Fourth, in assessing the model performance, we employed the receiver operating characteristic (ROC) curve and area under the curve (AUC) metrics, despite their known limitations^48^. Our findings indicate a 58.8% PPV and 92.4% NPV at the optimal cut-off, highlighting a significant proportion of false positives and a minimal occurrence of false negatives. Notably, the ROC–AUC framework does not show PPV or NPV^49^. Nonetheless, adhering to standard analytical practices for binary classifiers, we utilized the conventional AUC metric since it continues to be the preferred evaluative standard^48^. Fifth, BPC18 was not included in the final model in our analysis, despite it being significantly associated with flail chest in the univariable analysis. However, the univariable result implies that flail motion would be associated with greater intrathoracic energy dispersion, resulting in more significant pulmonary contusion (and impaired gas exchange). Further research into this issue is needed. Sixth, in our cohort, prolonged hospitalization durations might be attributed to the excellent health insurance system and the relatively low financial burden of hospital stays in our country. Therefore, the length of hospital stay may not serve as a reliable metric for assessing disease severity in this context. Finally, we did not conduct external validation. Overall, future multicenter trials and external validation are warranted.

## Conclusion

Our study suggests a novel nomogram for predicting paradoxical chest wall motion in patients with flail segments in traumatic rib fracture. The flail segment was a necessary but not sufficient condition for paradoxical chest wall motion. The number of rib fractures and Grade III segmental rib fractures were significant risk factors for flail motion, while the location of the rib fracture was also a significant risk factor. Future large-scale prospective studies are warranted to estimate the exact effect size.

### Supplementary Information

Supplementary Information.

## Data Availability

The datasets used and/or analysed during the current study available from the corresponding author on reasonable request due to legal or ethical restriction.

## Abbreviations

CT: Computed tomography
ISS: Injury severity score
AIS: Abbreviated injury scale
RFX: Patterns of rib fractures
PC: Pulmonary contusion
ED: Emergency department
BPC18: Blunt pulmonary contusion score
TTSS: Thorax Trauma Severity Score
RFS: Rib Fracture Score
CTS: Chest trauma score
IQR: Interquartile range (IQR)
LASSO: Least absolute shrinkage and selection operator
MLR: Multivariable logistic regression
ROC: Receiver operator characteristic
AUROC: Area under the ROC curve
MV: Mechanical ventilation
SSRF: Surgical stabilization of rib fractures
cOR: Crude odds ratio
aOR: Adjusted odds ratio
CI: Confidence interval
ICU: Intensive care unit
LOS: Length of stay
