# Supplementary material for: Novel nomogram for predicting paradoxical chest wall movement in patients with flail segment of traumatic rib fracture: a retrospective cohort study
Source: Sci Rep. 2023 Nov 20;13:20251. doi: 10.1038/s41598-023-47700-w (PMC10662329; doi:10.1038/s41598-023-47700-w)
Supplement: Supplementary file 1 — Supplementary Information. [file 41598_2023_47700_MOESM1_ESM.pdf]

Supplementary Figure 1. Anatomic boundaries of the rib fractures according to the anterior and posterior axillary lines.

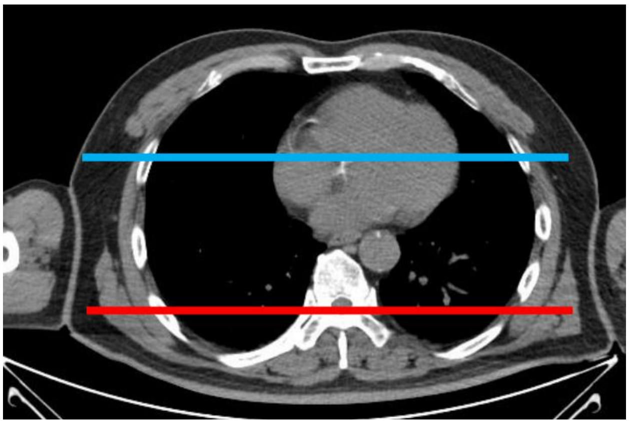

Supplementary Figure 2. An example of the primary fracture line. A posterolateral flail segment on the left chest wall, with more severe displacement in the posterior portion. The primary fracture line is located posteriorly in the chest wall of the patient.

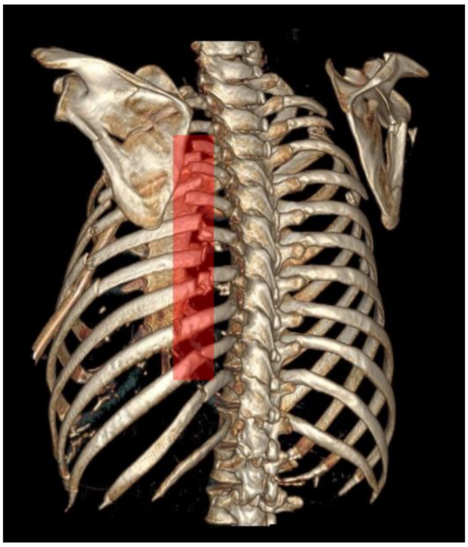

Supplementary Table 1. Compilation of the scoring system tables presented in each study. A: TTSS [26]; B: RFS [27]; C: CTS [28]; D: RibScore [29]

(A)

Thoracic Trauma Severity Score

Grade	PaO2/FiO2	Rib Fractures	Contusion	Pleural involvement	Age	Points
0	>400	0	None	None	<30	0
I	300-400	1-3	1 lobe, unilateral	PT	30-41	1
II	200-300	3-6	1 lobe, bilateral or 2 lobes unilateral	HT/HPT unilateral	42-54	2
III	150-200	>3 bilateral	<2 lobes bilateral	HT/HPT bilateral	55-70	3
IV	<150	Flail chest	≥2 lobes bilateral	Tension pneumothorax	>70	5

PT: pneumothorax; HT: hemothorax; HPT; hemopneumothorax
A minimum value of 0 points and a maximum value of 25 points can be achieved.

(B)

Rib Fracture Score

**Rib Fracture Score = (Breaks x sides) + Age factor**	
Breaks	Number of fractures
Sides	Unilateral = 1, bilateral = 2
Age factor	
0	<50
1	51-60
2	61-70
3	71-80
4	>80

(C)

Chest Scoring system

**Age Score**	
1	<45
2	45-65

3	>65
**Pulmonary contusion score**	
0	None
1	Unilateral minor
2	Bilateral minor
3	Unilateral major
4	Bilateral major
**Rib score**	
1	<3
2	3-5
3	>5
**Bilateral rib fractures**	
0	No
2	Yes

(D)

RibScore

Variables	Yes	No
≥6 Rib fractures	1	0
Flail chest	1	0
Bilateral fractures	1	0
First rib fracture	1	0
≥3 Displaced rib fractures	1	0
Fracture in each anatomic area	1	0

A minimum value of 0 points and a maximum value of 6 points can be achieved.
